# SEM and Bacteriological Evidence of Laser-Activated Irrigation Compared to Ultrasonic-Activated Irrigation: A Pilot Study

**DOI:** 10.3390/dj13050195

**Published:** 2025-04-29

**Authors:** David E. Jaramillo, Ji W. Jeong, Zhen Shen, Enrico Divito

**Affiliations:** 1 Department of Endodontics, School of Dentistry, University of Texas Health Science Center at Houston, Houston, TX 77054, USA; ji.wook.jeong@uth.tmc.edu (J.W.J.); shzhenen@gmail.com (Z.S.); 2 Arizona Center for Laser Dentistry, Scottsdale, AZ 85255, USA

**Keywords:** laser, Er;Cr:YSG, ultrasonic irrigation, bacterial count, sodium hypochlorite

## Abstract

**Background:** Pulp tissue debridement and the eradication of microorganisms from an infected root canal system before obturation is a primary focus of endodontic treatment and the best predictor for the long-term success of endodontic treatment. **Objective:** The purpose of this in vitro laboratory study was to evaluate pulp tissue debridement and the disinfection efficacy of two different Er;Cr:YSGG laser units, with a 2790 nm wavelength, compared to ultrasonic-activated irrigation (UAI) in root canals infected with *Enterococcus faecalis*. **Methods:** Human non-infected mandibular first molars were extracted, disinfected, and cultured with *Enterococcus faecalis*. Different types of Er;Cr:YSGG laser irrigation and UAI were performed according to the manufacturers’ protocols. The teeth were then processed for bacteriological and SEM analyses. **Results:** The different laser-activated irrigation protocols showed multiple areas of remaining bacteria, biofilm, tissue, and thermal ablation. The laser fiber tips also displayed significant tip degradation after use, which might affect efficacy. **Conclusions:** In this in vitro study, laser-activated irrigation using Er;Cr:YSGG technology and UAI were inefficient in eliminating pulp tissue from difficult-to-reach areas and *Enterococcus faecalis* from infected root canals.

## 1. Introduction

Numerous clinical methods aim to disinfect and control root canal biofilm in endodontic treatment, given that bacterial presence is a known cause of treatment failure [1,2,3]. The eradication of microorganisms from infected root canals before obturation is not only a primary goal of endodontic treatment [3,4] but also a significant factor in predicting long-term therapeutic success [5]. The disinfection of root canals is clinically challenging primarily due to the specific locations where bacteria reside and their ability to multiply. The survival of bacteria is facilitated by their ability to colonize dentinal tubules, lateral canal ramifications, isthmuses, and other root canal irregularities [1,3]. Consequently, mechanical instrumentation alone is insufficient and must be supplemented with antimicrobial solutions to effectively reduce bacterial load [6,7,8]. Due to its ability to endure and adapt to challenging environments, the Gram-positive facultative anaerobe *Enterococcus faecalis* is commonly found surviving in endodontic infections and periapical lesions [8]. The use of sodium hypochlorite, chlorhexidine (gel or liquid), and other irrigation solutions has been shown to be effective in the reduction or elimination of *E. faecalis* from root canals and dentinal tubules [9]. The enhancement of irrigant efficacy has been pursued through diverse methodologies, including the modulation of the concentration and temperature, the incorporation of surfactants, and the application of agitation protocols [10]. While traditional chemo-mechanical methods yield satisfactory endodontic results, studies indicate that incorporating lasers with irrigants can enhance outcomes in challenging cases [1,11,12].

Ultrasonic technology significantly improves root canal irrigation through cavitation and acoustic streaming, as supported by established research [13]. This in vitro study evaluated the disinfection efficacy of two distinct erbium/chromium/yttrium–scandium–gallium–garnet (Er;Cr:YSGG) lasers in comparison to ultrasonic-activated irrigation (UAI) in root canals inoculated with *E. faecalis*.

## 2. Material and Methods

**Figure 1:**
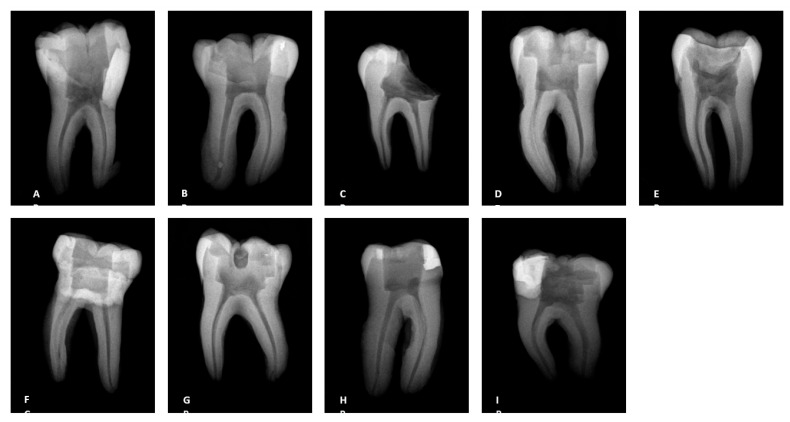
Radiographs of the teeth treated with three different irrigation protocols. (**A**–**C**) Laser #1 (NM), (**D**–**F**) mid-root laser #2 (CA), and (**G**–**I**) UAI.

A total of 32 human non-infected mandibular first molars extracted for therapeutic reasons were collected from patients presenting to the UTSHOD urgent care clinic. After an initial evaluation and the offer of treatment options for their dental condition, patients who elected to have tooth extractions were included in this study and provided consent. Following extraction, digital radiographs were obtained of each tooth (Figure 1). Tooth canal calcification and curvature were assessed, with root canal curvature classified according to the Schneider classification [14]. Roots with a curvature of less than 20 degrees were selected for inclusion in the study.

For the consistency of this study, teeth with canal obliteration, fractured roots, internal or external resorption, complex root anatomy, and a history of previous endodontic treatment were excluded from this study.

The selected teeth were cleaned to remove the soft tissue and any other material adhering to the outside of the teeth. The teeth were stored in a phosphate-buffered saline solution and frozen at −80 °C until use. Using a high-speed handpiece and a sterile #3 round carbide bur with water spray, access cavities were created. The DG-16 endodontic explorer was used to identify root canal orifices. The working length was determined with a #10 K-file (Kerr, Brea, CA, USA) by reducing the length where the file emerged from the apical foramen by 1 mm.

Root canals were prepared using a standard sequence of 0.04 taper rotary files, culminating in a 30/.04 Vortex Blue^®^ file (Charlotte, NC, USA)

After each file, the root canals were irrigated with 1 mL of 6% NaOCl using a syringe with a 30-gauge side-vented needle. Once the canals were prepared, NaOCl was inactivated with a 5% sodium thiosulfate solution for 5 min. The canals were then irrigated with 5 mL of 17% ethylenediaminetetraacetic acid (EDTA) for 5 min. The teeth were then placed in a glass flask and sterilized using an autoclave.

For *Enterococcus faecalis* biofilm formation, a 16 h culture of *E. faecalis* ATCC 4083 in 1 mL of brain heart infusion broth (BHI) was followed by inoculation into 5 mL of fresh BHI with 2% glucose.

The molars were placed individually into a 50 mL conical test tube with 10 mL of fresh BHI and inoculated with 100 µL of the suspension of the cultured E. faecalis. The molars were incubated at 37 °C for four weeks. The BHI medium was replaced once a week for four weeks of incubation time. The ability to colonize the root canal was verified by the 0.5 McFarland turbidity standard model and by observing the root canal surface of an untreated molar using SEM analysis.

### 2.1. Preparation of Laser Systems

Before use, each laser system—a smaller EdgePro unit (Albuquerque, NM, USA) and a larger Waterlase unit (Foothill Ranch, CA, USA)—was inspected as per the user manual. The mirror on the fiber optic was inspected for damage to ensure that it was in good condition. The mirror in the handpiece was inspected for damage to ensure it was in good condition. Each fiber tip was inspected as per the user manual. A different new tip was used for each tooth. Before and after the use of each new tip, the output power was measured using a Power Max 500D (Molectron, Portland, OR, USA). The expected power output at the fiber tip was calculated considering a 20% power loss during delivery.

### 2.2. Laser-Activated Irrigation (LAI) Protocols

Two teeth were not treated by LAI and ultrasonic irrigation and were placed into a vial containing a 10% formalin solution. It served as a positive control to verify, by means of SEM, that an E. faecalis biofilm was established in the root canal system using the inoculation procedure described above. The second tooth was histologically sectioned and stained with H&E and Gram to verify the preservation of pulp tissue and bacterial growth.

Thirty teeth were randomly divided into six groups (n = 5). Each group underwent a different laser-activated irrigation protocol or ultrasonic-activated irrigation (UAI): (1) 0.5% NaOCl + Laser #1, (2) 3% NaOCl + 17% EDTA + Laser #1, (3) 3% NaOCl + 17% EDTA + Laser #2, (4) 0.5% NaOCl + Laser #2, (5) 0.5% NaOCl + ultrasonic tip, and (6) 3% NaOCl + ultrasonic tip (Table 1). These teeth were examined using SEM analysis.

For 0.5% NaOCl + EdgePro, an RFT (275) tip and Waterlase Tip 2 (320) were used. The laser settings were 10 Hz, 1 W, 100 mJ/pulse, and a 60 µs pulse length, with the air/water turned off. These custom settings were from the laser disinfection section of the EdgePro Radial Apical Cleansing Protocol [13]. The settings were programmed into EdgePro as previously described [15]. The pulp chamber of the tooth was flooded with 0.5% NaOCl, and a number 2 tip (320 µM) was placed above each root canal orifice and activated for 30 s while holding the tip in place. After a 30 s rest period, the tip was activated again for 30 s. Then, the chamber was flooded with distilled water, and the tip was activated for 30 s over each canal orifice. After the laser-activated irrigation, the teeth were placed into a vial containing a 10% formalin solution.

For 3% NaOCl + 17% EDTA + EdgePro, for the debridement and disinfection step, the laser settings were 50 Hz, 1.25 W, 25 mJ/pulse, and a 60 µs pulse length, with the air/water turned off. The pulp chamber of the tooth was flooded with 3% NaOCl. Using consistent irrigation with 3% NaOCl, a number 2 tip (320 µM) was placed mid-root; then, the laser was activated, and the fiber tip was withdrawn at 1 mm/s. The same step was repeated three times per canal. Next, the pulp chamber of the tooth was flooded with 17% EDTA. With consistent EDTA irrigation, a number 2 tip (320 µM) was placed mid-root; then, the laser was activated, and the fiber tip was withdrawn at 1 mm/s. The same step was repeated three times per canal. For the post-rinse cycle, he pulp chamber of the tooth was flooded with water, and a number 2 tip (320 µM) was placed in the pulp chamber; the laser was activated for 30 s using the post-rinse settings (20 Hz, 1.50 W, 75 mJ/ pulse, 60 µs pulse length, 20% air, and 90% water). After the laser-activated irrigation, the teeth were placed into a vial containing a 10% formalin solution.

For 3% NaOCl + 17% EDTA + Waterlase, the laser settings were 50 Hz, 1.25 W, 25 mJ/ pulse, and a 60 µs pulse length, with the air/water turned off. The pulp chamber of the tooth was flooded with 3% NaOCl, and an RFT2 tip was placed 2 mm short of the working length; the laser was activated, and the fiber tip was withdrawn at 2 mm/s. There were three passes per canal. Then, the pulp chamber of the tooth was flooded with 17% EDTA, and an RFT2 tip was placed 2 mm short of the working length; the laser was activated, and the fiber tip was withdrawn at 2 mm/s. There were three passes per canal. The pulp chamber of the tooth was flooded with water, and an RFT2 tip was placed 2 mm short of the working length; the laser was activated, and the fiber tip was withdrawn at 2 mm/s. There were three passes per canal. After the laser-activated irrigation, the teeth were placed into a vial containing a 10% formalin solution.

For the 0.5% and 3% NaOCl Irrisafe UAI group, the tip was located 1 mm short of the working length and activated at 25% power, running three cycles of 20 s alternating NaOCl, EDTA, and NaOCl.

A total of 5 mL of NaOCl and 2 mL of EDTA were used individually in each experimental tooth.

## 3. Bacteriological Samples

NaOCl was inactivated with the irrigation of 1 mL of 5% sodium thiosulfate for 1 min, followed by 0.5 mL of sterile saline solution. A sterile size 35 Hedstrom file (Charlotte, NC, USA) was used to collect dentinal shavings by planing the canal walls. A second bacterial sample (S2) was collected with two medium sterile paper points. The paper points were left in the canal to absorb the solution for 1 min each. Both the paper points and the Hedstrom file were placed in a micro-Eppendorf test tube filled with 0.5 mL of PBS. The teeth were then stored in formalin for SEM evaluation.

### 3.1. CFU Bacterial Analysis

Sterile micro-Eppendorf tubes were filled with 90 uL of PBS per sample collected. The micro-Eppendorf tubes with the samples were then vortexed, and 10 uL was collected from the original sample and placed into another micro-Eppendorf tube. This serial dilution continued up to 10^−5^, and CFU counts were then completed after 24 h, and statistical analysis was completed thereafter.

### 3.2. Radiographs

Using a Pennwalt^®^ Intrex JC03772 X-ray machine (Pennwalt, Mumbai, India), radiographs were taken of each tooth before splitting the teeth for SEM.

### 3.3. Scanning Electron Microscopy (SEM)

Before the roots were separated, the crowns of each specimen were removed, and each root was split in half with the use of a low-speed diamond disk under a water coolant. The split roots were placed in glutaraldehyde for 48 h, and then samples were dehydrated using the serial ethanol dehydration technique. Roots were mounted on an SEM tab with carbon conductive tape and sputter coated with a 60:40 gold/palladium target using a Sputter Coater (Microsystems, Northbrook, IL, USA). The samples were imaged using SEM with a FEI™ Nova NanoSEM 230 (FEI Company, Census Bureau, OR, USA). Images were taken using between 50× and 20,000× magnification. One exact visualization point was selected in each of the three regions of the root (coronal, middle, and apical) within the root canal lumen and isthmus area. When lateral canals were detected, they were explored, regardless of their position for exploratory purposes.

### 3.4. Stereomicroscope Images

For each LAI protocol, the laser fiber tip was saved. Each fiber tip was examined with a Nikon SMZ800N stereomicroscope (Melville, NY, USA), and images were taken and processed (Imaging software NIS/Elements Version 4.13) under the same 5× magnification to document the fiber tip condition after use.

### 3.5. Statistical Analysis

All analyses were performed using R statistical software (R Core Team 2022). Statistical analysis was conducted using one-way ANOVA to determine whether there were significant differences among groups.

## 4. Results

### 4.1. Bacteriological Samples

The *p* value for the difference before and after the irrigation technique and between NaOCl concentrations demonstrated that there was no difference between either treatment performed.

The tables (Table 2, Table 3 and Table 4) show no effect among irrigation techniques or NaOCl concentrations.

### 4.2. SEM Analyses

**Figure 2:**
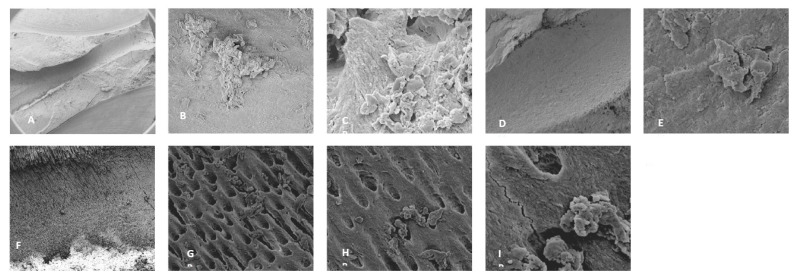
Sample SEM from all regions of the mesial canal treated with 0.5% NaOCl and Laser #1 (NM). Apical region: (**A**) 80× magnification; (**B**) at 3000× magnification, debris was observed; (**C**) at 13,000× magnification, debris and bacteria were observed. Middle region: (**D**) 500× magnification, and (**E**) at 11,000× magnification, debris and a smear layer were observed. Coronal region: (**F**) 400× magnification; (**G**) at 4500× magnification, debris and bacteria were observed; (**H**) at 7000× magnification, debris was observed; (**I**) at 7000× magnification, debris and bacteria were observed.

**Figure 3:**
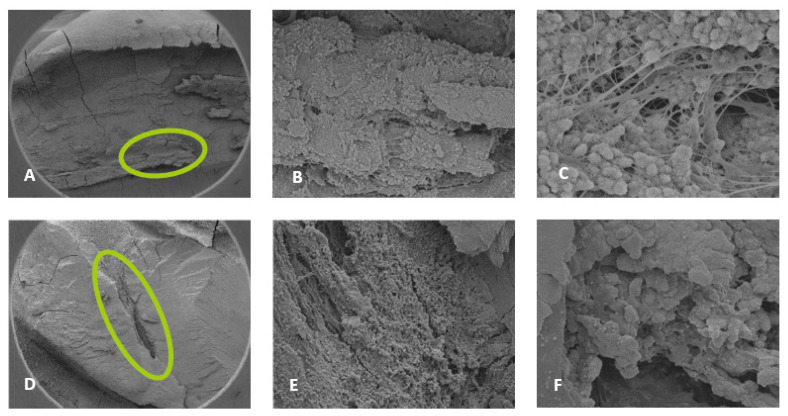
Sample SEM images from complex areas of the mesial canals treated with 0.5% NaOCl and Laser #1 (NM). (**A**) 70× magnification of a mesial canal and isthmus. (**B**) At 300× magnification, pulp tissue, bacteria, and biofilm were observed. (**C**) At 13,000× magnification, pulp tissue fibers, bacteria, and biofilm were observed. (**D**) At 75× magnification, a middle mesial with tissue was observed in sample 3. (**E**) At 2000× magnification, pulp tissue and a bacterial biofilm were observed. (**F**) At 2500× magnification, tissue and bacteria were observed.

**Figure 4:**
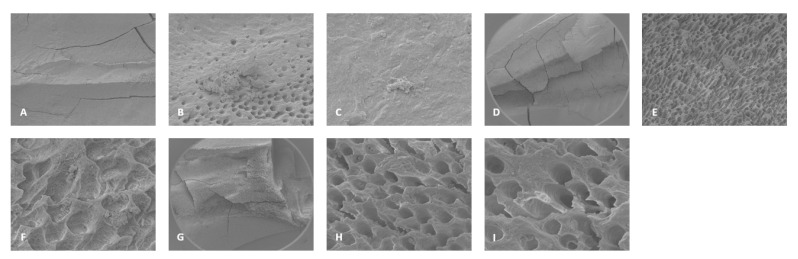
Representative SEM images of distal canals. Apical regions: (**A**) 300× magnification; (**B**) at 3000× magnification, debris was observed, and (**C**) at 10,000× magnification, debris and bacteria were observed. Middle region: (**D**) 70× magnification; (**E**) at 2000× magnification, debris was observed; (**F**) at 11,000× magnification, debris was observed in the dentinal tubules. Coronal region: (**G**) at 70× magnification, ablation was observed; (**H**) at 9000× magnification, debris and damaged dentinal tubules were observed; and (**I**) at 12,000× magnification, debris and damaged dentinal tubules were observed.

**Figure 5:**
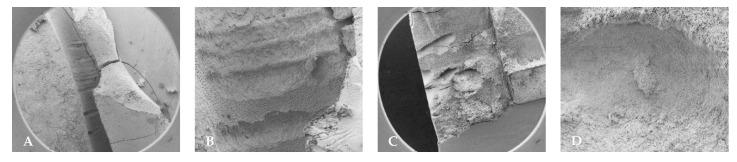
Sample SEM images from the root canal and pulp chamber showing areas of ablation. (**A**) Root canal at 70× magnification and (**B**) at 500× magnification; (**C**) pulp chamber at 78× magnification and (**D**) at 600× magnification.

For the 0.5% NaOCl + Laser #1 samples, some debris and bacteria were observed in the mesial canals in the apical, middle, and coronal regions of all three teeth (Figure 2). In addition, tissue, bacteria, and well-organized biofilm were observed in the isthmus region of the mesial canals of all three teeth (Figure 3A–C). In sample #3, significant amounts of tissue and bacteria were observed in the middle region of the mesial canal (Figure 3D–F). In the distal canals, some debris and bacteria were observed (Figure 4). Areas of ablation were also observed in the pulp chamber of all three samples (Figure 5).

**Figure 6:**
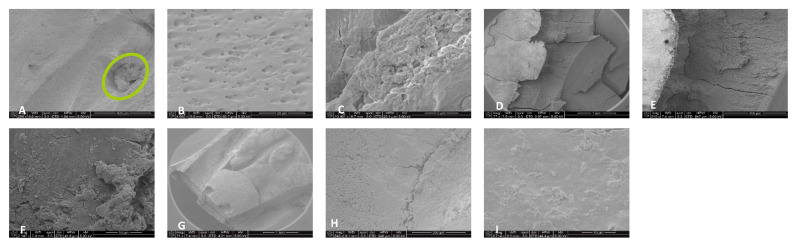
Sample SEM images of mesial canals treated with 3% NaOCl + 17% EDTA + Laser #1. Apical region: (**A**) at 300× magnification, a lateral canal with tissue was observed; (**B**) at 3000× magnification, a few bacteria were observed; (**C**) at 13,000× magnification, debris and bacteria were observed. Middle region: (**D**) 70× magnification; (**E**) at 300× magnification, thermal damage was observed; (**F**) at 7000× magnification, debris and bacteria were observed. Coronal region: (**G**) 70× magnification; (**H**) at 500× magnification, ablation was observed; (**I**) at 7000× magnification, debris, bacteria, and the absence of open dentinal tubules were observed.

**Figure 7:**
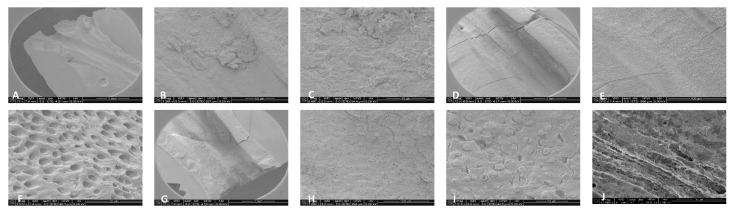
Representative images of distal canals treated with 3% NaOCl + 17% EDTA + Laser #1. Apical region: (**A**) 71× magnification; (**B**) at 1200× magnification, debris was observed in the lateral canal; (**C**) at 8000× magnification, debris and bacteria were observed. Middle region: (**D**) 70× magnification; (**E**) at 300× magnification, thermal damage was observed; (**F**) at 6000× magnification, debris and bacteria were observed. Coronal region: (**G**) 70× magnification; (**H**) 1000× magnification; (**I**) at 7000× magnification, thermal damage was observed; (**J**) at 6000× magnification, UAI and bacteria within dentinal tubules were observed in the UAI group.

**Figure 8:**
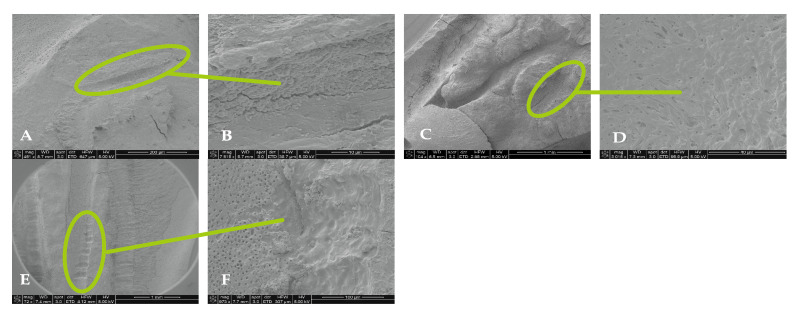
Representative images of the isthmus areas and ablation areas for teeth treated with 3% NaOCl + 17% EDTA + Laser #1. Apical region: (**A**) 500× magnification; (**B**) at 8000× magnification, tissue debris and bacteria were observed in the isthmus. Coronal region: (**C**) 100× magnification; (**D**) at 3000× magnification, collagen fibers and bacteria were observed. Middle region: (**E**) at 70× magnification, areas of ablation were observed; and (**F**) at 1000× magnification, some areas of the dentinal tubules appeared to be melted together, indicating thermal damage due to ablation.

**Figure 9:**
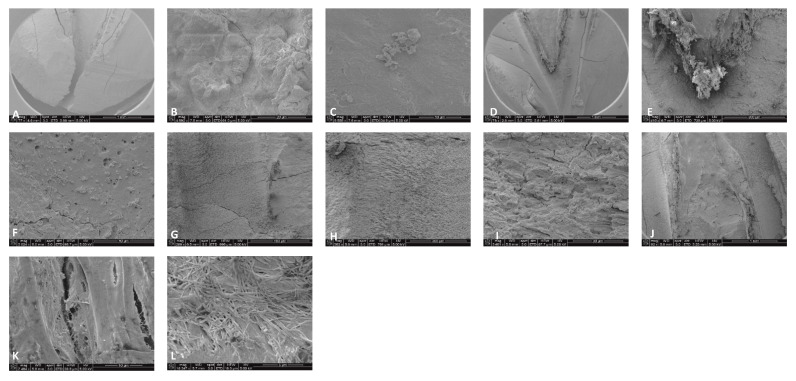
Representative images of the isthmus areas and lateral canals for teeth treated with 3% NaOCl + 17% EDTA + Laser #2. Apical region: (**A**) 80× magnification; (**B**) at 5000×, there were areas without open dentinal tubules; (**C**) at 9000×, debris and a few bacteria were observed (no dentinal tubules visible). Middle region: (**D**) 70× magnification; (**E**) at 400× magnification, tissue was observed in the isthmus; (**F**) at 3000× magnification, debris and bacteria were observed; (**G**) 70× magnification; (**H**) at 400× magnification, areas of ablation were observed; (**I**) at 3500× magnification, debris, bacteria, and biofilm were observed. Coronal region: (**J**) 90× magnification; (**K**) at 7500× magnification, tissue and bacteria were observed; (**L**) at 16,000× magnification, tissue, bacteria, and collagen-like structures were observed.

**Figure 10:**
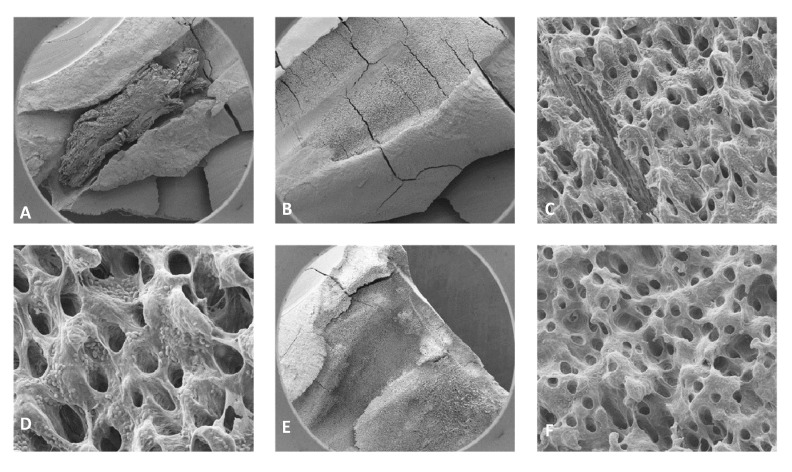
Sample SEM images of a distal canal treated with Laser #2. (**A**) 70× magnification of the apical region showing tissue. (**B**) 80× magnification of the middle region of the distal canal. (**C**) At 3500× magnification, biofilm and bacteria were observed. (**D**) At 7500× magnification, biofilm and bacteria were observed. (**E**) 70× magnification of the coronal region of the distal canal showing areas of ablation. (**F**) At 3500× magnification, bacteria were observed in the predentin area.

For 3% NaOCl + 17% EDTA + Laser #2 (CA) and UAI, some debris and bacteria were observed in the apical, middle, and coronal regions of the mesial and distal canals (Figure 6 and Figure 7). Areas of ablation were observed in all regions of the canal (Figure 8). In some areas, there were no open dentinal tubules, and some dentinal tubules appeared to be melted together or occluded, indicating possible thermal damage. Additionally, tissue, debris, and bacteria were observed in the isthmus areas and lateral canals in the apical, middle, and coronal regions of the mesial and distal canals (Figure 6, Figure 9 and Figure 10).

### 4.3. Stereomicroscope Images

**Figure 11:**
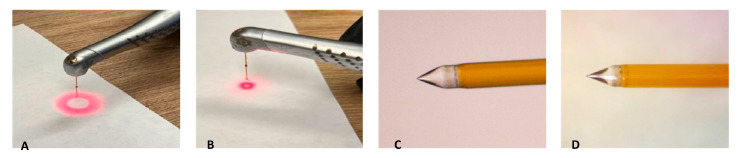
(**A**) Fiber inspection shows a halo, which indicates the fiber is good. (**B**) Fiber inspection shows a diffuse dot, which indicates that the tip is broken (after single use). (**C**) New RFT2 tip from the Waterlase unit, and (**D**) Tip 2 from EdgePro.

**Figure 12:**
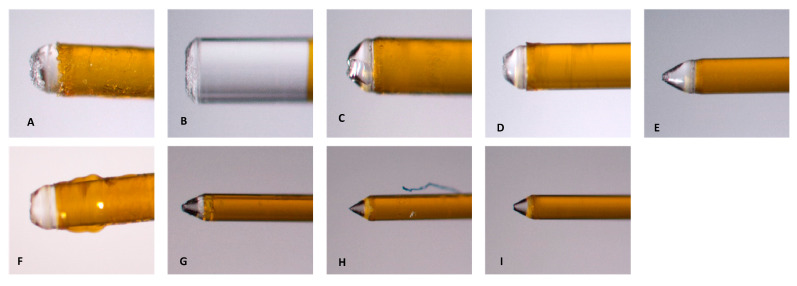
Images of the fiber tips used in the experiments. (**A**–**C**) The three tips used in the 0.5% NaOCl + EdgePro group. (**D**–**F**) The three tips used in the 3% NaOCl + 17% EDTA + EdgePro group. (**G**–**I**) The three tips used in the 3% NaOCl +17% EDTA + Waterlase group.

Prior to use, the fiber tips for Laser #1 and Laser #2 were inspected using the aiming beam, as per their respective manuals. Figure 11 shows an example of the shape of the aiming beam of a Laser #1 fiber tip before and after use, a new RFT2 fiber tip for Laser #2, and a new Tip 2 for Laser #1. Before use, the fiber tip showed the halo expected for a new tip (Figure 11A). After use, the halo was no longer evident (Figure 11B). For the 0.5% NaOCl + Laser #1 samples, the fiber tips showed that the radial tip was degraded and blunted (Figure 12A–C). For 3% NaOCl + 17% EDTA + Laser #1, the fiber tips showed that the radial tip was degraded and blunted (Figure 12D–F). For 3% NaOCl + 17% EDTA + Laser #2, the fiber tips showed minimal damage to the radial tip (Figure 12G–I).

## 5. Discussion

With the introduction of Laser #1, there is renewed interest in the performance of Er;Cr:YSGG lasers for root canal treatments. The disinfecting abilities of the two laser-activated irrigation (LAI) protocols that use the Er;Cr:YSGG laser were evaluated and compared to UAI by treating teeth inoculated with E. faecalis, which is frequently detected in root canal infections, and evaluating the teeth for the presence of bacteria, biofilm, tissue, debris, and a smear layer.

Use of the Laser #1 Radial Apical Cleansing Protocol for the cleansing and disinfection of root canal systems has been proposed [16], and it is reported to effectively remove bacteria and biofilm from canals, complex areas, and dentinal tubules. The same report claims that the protocol is safe and minimizes the chance of extrusion because the recommended concentration of NaOCl is low (0.5% NaOCl) and the fiber tip is in the chamber. This study used the laser disinfection step (0.5% NaOCl + Laser #1) from the above-recommended protocol to treat five mandibular first molars. Using SEM, the teeth were evaluated for the presence of bacteria, biofilm, tissue, debris, and a smear layer. In general, some debris and bacteria were observed throughout the canals, particularly in the apical region of the canals (Figure 3 and Figure 5). Although the protocol is designed to clean and disinfect isthmus areas, we observed tissue, bacteria, and biofilm in the isthmus areas between the mesial canals for the five treated teeth. In addition, one tooth had an uninstrumented middle mesial canal, in which we observed tissue, bacteria, and biofilm even though the protocol should have cleaned and disinfected uninstrumented canals such as this one [16]. Furthermore, evidence of ablation was observed in the pulp chamber of each laser-treated tooth, possibly due to the laser settings of 100 mJ/pulse. Previous reports indicated dentine may be ablated or otherwise damaged if exposed to 20 mJ/pulse or higher [17,18].

In this study, the solutions/mid-root protocol from the Laser #1 manual (denoted as 3% NaOCl + 17% EDTA + Laser #1) was used to treat five mandibular first molars. Based on the Laser #1 website, the recommended protocols “provide outstanding cleaning, debridement, disinfection by removing infected tissue, biofilm, and smear layer, killing up to 99% of bacteria commonly found in the root canal” [19]. In general, some tissue remnants were observed in the mesial and distal canals as well as areas of debris and bacteria. Tissue, debris, bacteria, and biofilm were observed in the isthmus areas and lateral canals, which are considered the more difficult areas to reach with irrigation systems. In addition, there were areas of ablation in the canals, including locations where the dentinal tubules appeared to look melted together. This possibly indicates that fluid needs to be maintained in the canals while activating the laser to help minimize the likelihood of thermal damage.

Laser #2 recommends the RAPIDENDO—Radial Apical Irrigation and Disinfection protocol for use with Laser #2. According to this protocol, the smear layer and biofilm are removed and the dentinal tubules are opened to facilitate deeper cleaning [20]. In general, this study observed debris and bacteria in the apical, middle, and coronal regions of the mesial and distal canals. In some cases, tissue was also observed in the apical region of the canals. Tissue, debris, bacteria, and biofilm were observed in the hard-to-reach areas, such as the isthmus areas and uninstrumented lateral canals, in all five treated teeth. Furthermore, bacteria were observed in all regions of one distal canal, which was very wide. This may indicate that further study is warranted to demonstrate that the laser action is effective in very wide canals. In addition, there were areas where no open dentinal tubules were observed, which possibly indicates that the smear layer had not been removed.

Ultrasonic-activated irrigation (UAI) refers to a technique used in the irrigation of the root canal system, utilizing ultrasonic energy that is employed to enhance the irrigation process. This technique [13] has gained widespread acceptance and is now commonly regarded as the standard of care in endodontics. Our findings align with the previous documented scientific literature.

Before all the experiments with LAI, the condition of the tip was evaluated using the system’s aiming beam, as per the manufacturer’s recommendations. Even though many laser users believe that the fiber tips are reusable [21], the aiming beam test performed as per the manufacturer’s manual and the images of the tips (Figure 12) after using them to treat a single mandibular first molar indicated that the tip was broken and was no longer performing optimally after one use.

All these SEM observations are supported by the bacteriological results from sampling the canals.

SEM allows for the high-resolution imaging of the surface of samples, providing detailed information about the morphology, structure, and spatial distribution of microorganisms. Using SEM, it is possible to observe the physical characteristics of microbial colonies and tissue remnants. On the other hand, bacteriological results from sampling the canals involve the collection and analysis of microbial samples from the canal system. By combining SEM observations with bacteriological results, it is possible to correlate the visual characteristics and the presence of microorganisms.

The purpose of the study was to investigate and compare the efficacy and efficiency of each technique. In this study, the small sample size (n = 5) may have influenced the statistical results, potentially limiting the ability to detect differences. Therefore, increasing the sample size in future studies is necessary.

## 6. Conclusions

In the present in vitro study, laser-activated irrigation using Er,Cr:YSGG technology and UAI were inefficient in eliminating *Enterococcus faecalis* from infected root canals, and tissue, debris, bacteria (dentinal tubules), and biofilm were observed in isthmus areas, lateral canals, and the uninstrumented areas of teeth after treatment using the manufacturer recommended protocols. Additional studies are planned to confirm these results.

## Figures and Tables

**Table 1:** Laser-activated and ultrasonic-activated irrigation protocols.

Group	Technique	Irrigation Protocol
1	EdgePro	0.5% Sodium hypochlorite
2	EdgePro	3% Sodium hypochlorite + 17% EDTA
3	Waterlase	3% Sodium hypochlorite + 17% EDTA
4	Waterlase	0.5% Sodium hypochlorite
5	UAI	0.5% Sodium hypochlorite
6	UAI	3% Sodium hypochlorite

**Table 2:** Anova Table.

	LR Chi sq	Df	Pr (>Chi sq)
Irrigation	1.48497	2	0.4759
NaOCl	0.05221	1	0.8193
Irrigation: NaOCl	0.49962	2	0.7789

**Table 3:** Mean variation among techniques and NaOCl concentrations.

	0.5%	3%
EdgePro	354,212.0	381,660.0
UA	328,920.8	44,959.4
WaterLase	487,995.4	584,121.4

**Table 4:** Standard deviation among techniques and NaOCl concentrations.

	0.5%	3%
EdgePro	752,692.3	741,913.43
UA	500,120.5	39,708.67
WaterLase	598,095.4	858,527.02

## Data Availability

The original contributions presented in this study are included in the article. Further inquiries can be directed to the corresponding author.
